# Surgical Reconstruction of a Left Ventricular Aneurysm Using an Extracellular Matrix Patch

**DOI:** 10.21470/1678-9741-2021-0045

**Published:** 2022

**Authors:** Igor Zivkovic, Vladimir Mihajlovic, Djordje Zdravkovic, Djordje Krstic, Srasa Krasic, Jelena Lesanovic, Miodrag Peric, Petar Milacic

**Affiliations:** 1 Department of Cardiac Surgery, Dedinje Cardiovascular Institute, Belgrade, Serbia.; 2 Department of Radiology, Dedinje Cardiovascular Institute, Belgrade, Serbia.; 3 Department of Cardiology, Mother and Child Health Institute of Serbia, Belgrade, Serbia.; 4 Department of Anesthesiology, Dedinje Cardiovascular Institute, Belgrade, Serbia.; 5 School of Medicine, University of Belgrade, Belgrade, Serbia.

**Keywords:** Left Ventricle, Heart Aneurysm, Extracellular Matrix, Stroke Volume

## Abstract

The left ventricular aneurysm is a pathological condition defined as an akinetic or dyskinetic area of the left ventricle (LV) wall associated with reduced ejection fraction. The most common surgical technique to reconstruct a left ventricular aneurysm is endoventricular patch plasty (Dor procedure). In this case, endoventricular reconstruction of the left ventricular aneurysm using a double-layer extracellular matrix was performed.

**Table:** 

Abbreviations, acronyms & symbols	
CK-MB	= Creatine kinase-MB
CT	= Computed tomography
ECG	= Electrocardiogram
LV	= Left ventricle
LVESD	= Left ventricular end-systolic diameter
LVEDD	= Left ventricular end-diastolic diameter
STEMI	= ST-elevation myocardial infarction

## INTRODUCTION

The left ventricular aneurysm is a pathological condition defined as an akinetic or dyskinetic area of the left ventricle (LV) wall associated with reduced ejection fraction^[1]^. One of the most common surgical solutions for reconstructing a sizable left ventricle aneurysm is the endoventricular patch technique (Dor procedure). It improves the ventricle size and geometry, reduces wall stress and paradoxical movement, and increases the LV ejection fraction^[2]^. Commonly used materials as a patch for reconstructing the LV aneurysm are Dacron, bovine pericardium and Teflon. We present our experience in reconstructing the apical LV aneurysm using a double-layer extracellular matrix (ProxiCor for cardiac tissue repair, Aziyo Biologics, Roswell, GA, USA) endoventricular patch.

A 61-year-old male was admitted to the hospital due to dyspnea symptoms, sharp chest pain during physical activity and profuse sweating. He had a history of ST-elevation myocardial infarction (STEMI) two years earlier, when he underwent primary percutaneous coronary intervention with implantation of two drug-eluting stents in the left anterior descending artery. The postprocedural period was uneventful.

**Fig. 1:**
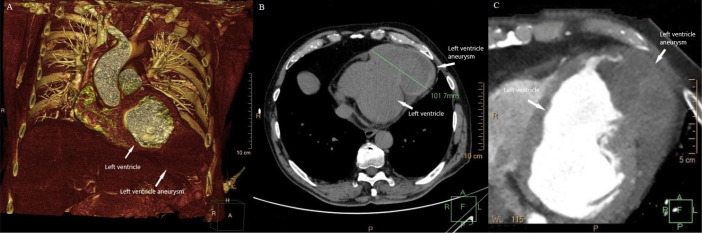
ECG-gated cardiac CT. (A) 3D volume rendering reconstruction - well-defined aneurysmal sac in the antero-inferior lateral aspect of the left ventricle. (B-C) Axial view of unenhanced and contrast-enhanced phase - active contrast extravasation into the aneurysmal sac.

Cardio-specific enzymes (high-sensitivity troponin T and CK-MB) were in the referent range at admission to the hospital. Transthoracic echocardiography (apical four-chamber view) revealed a dyskinetic apical segment of the left ventricle (LV) and compression of the lateral apical wall and apical segment by an organized thrombotic mass measuring 110 × 47mm (Video 1). Ejection fraction was 25%, with akinesis of inferior, posterior and apical segments of the LV wall. The LV diameters were normal (LVESD 40 mm, LVEDD 55 mm). Multislice computed tomography revealed a giant aneurysm and thrombotic formation in a projection of the left ventricular apex measuring 101 × 63 mm with a cranial-caudal diameter of 65 mm (Figure 1A, B and C). Preoperative coronary angiography did not reveal significant coronary or in-stent stenosis.

**Video 1:**
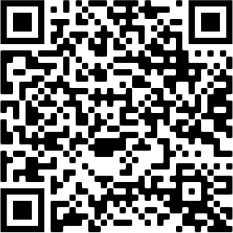
Apical four-chamber view of transthoracic echocardiography revealed anterolateral and apical giant aneurysm and thrombotic formation.

## TECHNIQUE

**Fig. 2:**
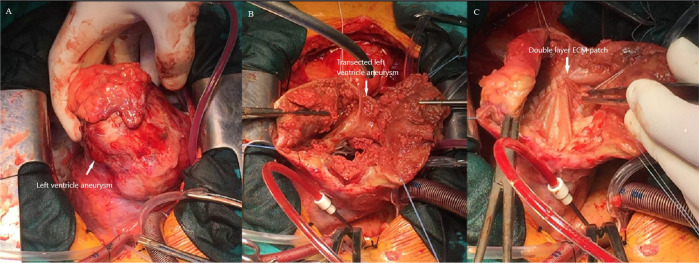
Surgical reconstruction of the left ventricular aneurysm. (A) Surgical view of the giant left ventricular aneurysm. (B) The aneurysmal cavity transected after thrombus debridement and detection of the junction between scar and viable myocardium. (C) Double-layer extracellular matrix sutured on the aneurysmal rim using polypropylene over and over the suture. ECM=extracellular matrix.

The surgical procedure was performed through a median sternotomy. Cardio-pulmonary bypass was instituted by ascending aorta and right atrial appendage cannulation. A blood cardioplegic solution was used to arrest the heart. A giant LV apical aneurysm was found after extensive dissection of the pericardial adhesions (Figure 2A). Longitudinal incision of the aneurysm and debridement of the thrombus were performed (Figure 2B). The rim of the aneurysm (the junction between the scar and viable myocardium around the entire circumference of the aneurysm) was detected, and the rest of the aneurismal tissue was trimmed to allow closure of the aneurysmal wall over the endoventricular patch. The ventricular wall defect was reconstructed using a double-layer extracellular matrix (ProxiCor) patch cut to a size sufficient to restore the average ventricular size (Figure 2C). The remaining aneurysmal wall was closed over an extracellular matrix using a two Teflon felts with double row 2-0 polypropylene running suture. There was no bleeding after surgical reconstruction.

During the early postoperative period, the patient had a short episode of disorientation, which was medically treated. The remainder of the postoperative period was uneventful, and the patient was discharged from the hospital on the 7^th^ postoperative day.

**Fig. 3:**
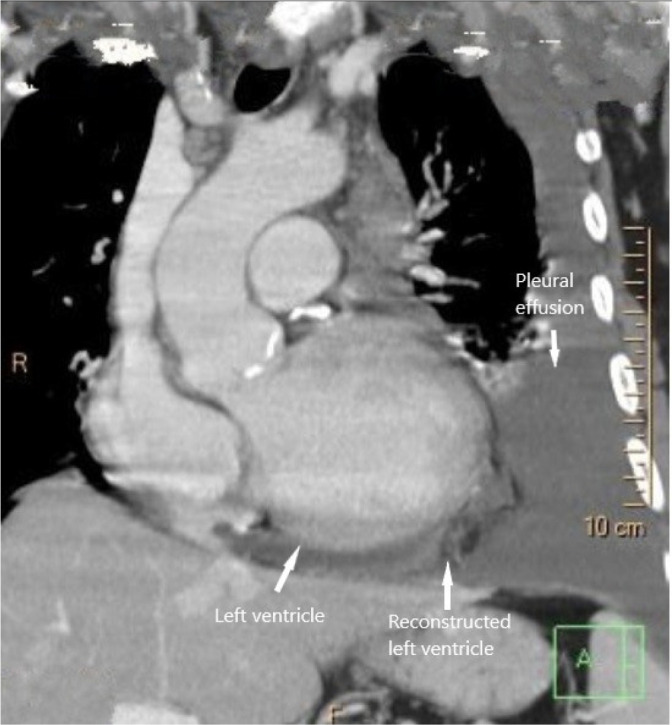
ECG-gated cardiac CT. Postoperative imaging showing extirpated aneurysmal sac with normal postoperative anatomy of the reconstructed left ventricle and postoperative pleural effusion.

The control ECG-gated CT scan performed 30 days after the surgical procedure showed left pleural effusion without new pseudoaneurysm and local complications of the reconstructed ventricle (Figure 3). The 600 ml serosanguinous pleural effusion was evacuated.

## DISCUSSION

Less than 5% of patients after transmural myocardial infarction develop LV aneurysm^[3]^. Progressive heart failure, persistent angina, thromboembolism and uncontrolled recurrent ventricular arrhythmias indicate surgical reconstruction^[4]^. The primary aim of surgical treatment is LV “reshaping”, which improves cardiac function, symptoms and life expectancy. In the late 1980s, cardiac surgeon Vincent Dor developed the new surgical concept of LV aneurysm reconstruction using an endoventricular Dacron or pericardium patch. This technique has four advantages: it excludes the akinetic segment of the interventricular septum; restores the physiological geometry of the ventricular cavity; preserves the left anterior descending artery for coronary bypass; and eliminates the need for external prosthetic materials, which can cause persistent pericardial adhesions^[1]^. The routine reconstruction of the LV wall is performed using an autologous, homologous or synthetic material. The main disadvantages of autologous or bovine pericardium are postprocedural fibrosis, thickening, and calcification. They may result in pseudoaneurysm formation, tissue retraction and rupture after the surgical repair^[5]^. In addition, a synthetic material such as woven nylon (Dacron) could promote reactive inflammation and increase the risk of endocarditis^[5,6]^. After implantation, the material becomes incorporated by fibrotic encapsulation and cannot fully restore the functionality of the regional tissue^[7]^.

The novel biological material, an extracellular matrix, has been introduced in surgery due to its positive characteristics (durability, easy handling during surgery, and resistance to calcification, thickening or retraction), which has been increasingly used in recent years. Tissue remodelling and regeneration are shown in preclinical experiments and clinical practice. Pathohistological examination revealed that the extracellular matrix was replaced by organized collagen, neovascularization and reendothelization^[5-8]^. Yanagawa et al.^[9]^ showed initial experience with interventricular repair using extracellular matrix. However, the short-term period (207±211 days) was uneventful. Patients with congenital heart disease who underwent cardiac surgery to reconstruct a pulmonary artery, right ventricular outflow tract, ascending aorta, and heart valve had no evidence of patch-related complications in the medium term^[10]^. There is no evidence of long-term results after LV reconstruction using an extracellular matrix patch. McCready et al. present long-term results after carotid artery reconstruction using multilayer extracellular patch to increase 0.3% of new formed pseudoaneurysms during 72 of months follow-up^[11]^.

Because of these characteristics, we decided to use the extracellular matrix as an endoventricular patch to reconstruct the LV. In addition, due to the risk of postoperative rupture and pseudoaneurysm formation, which were described as an early postoperative complication^[12]^, we performed a double-layer Proxicor patch to reconstruct the LV aneurysm.

## CONCLUSION

The extracellular matrix can be used as a substitute for a Dacron, bovine and autologous pericardium in the endoventricular reconstruction of the left ventricle aneurysm. In addition, the double-layer surgical technique could be implemented to increase the patch strength and prevent potential adverse events.

**Table:** 

Authors' roles & responsibilities			
IZ	Substantial contributions to the conception or design of the work; or the acquisition, analysis, or interpretation of data for the work; drafting the work or revising it critically for important intellectual content; agreement to be accountable for all aspects of the work in ensuring that questions related to the accuracy or integrity of any part of the work are appropriately investigated and resolved; final approval of the version to be published	SK	Substantial contributions to the conception or design of the work; or the acquisition, analysis, or interpretation of data for the work; drafting the work or revising it critically for important intellectual content; agreement to be accountable for all aspects of the work in ensuring that questions related to the accuracy or integrity of any part of the work are appropriately investigated and resolved; final approval of the version to be published
VM	Substantial contributions to the conception or design of the work; or the acquisition, analysis, or interpretation of data for the work; drafting the work or revising it critically for important intellectual content; agreement to be accountable for all aspects of the work in ensuring that questions related to the accuracy or integrity of any part of the work are appropriately investigated and resolved; final approval of the version to be published	JL	Substantial contributions to the conception or design of the work; or the acquisition, analysis, or interpretation of data for the work; drafting the work or revising it critically for important intellectual content; agreement to be accountable for all aspects of the work in ensuring that questions related to the accuracy or integrity of any part of the work are appropriately investigated and resolved; final approval of the version to be published
DZ	Substantial contributions to the conception or design of the work; or the acquisition, analysis, or interpretation of data for the work; drafting the work or revising it critically for important intellectual content; agreement to be accountable for all aspects of the work in ensuring that questions related to the accuracy or integrity of any part of the work are appropriately investigated and resolved; final approval of the version to be published	MP	Substantial contributions to the conception or design of the work; or the acquisition, analysis, or interpretation of data for the work; drafting the work or revising it critically for important intellectual content; agreement to be accountable for all aspects of the work in ensuring that questions related to the accuracy or integrity of any part of the work are appropriately investigated and resolved; final approval of the version to be published
DK	Substantial contributions to the conception or design of the work; or the acquisition, analysis, or interpretation of data for the work; drafting the work or revising it critically for important intellectual content; agreement to be accountable for all aspects of the work in ensuring that questions related to the accuracy or integrity of any part of the work are appropriately investigated and resolved; final approval of the version to be published	PM	Substantial contributions to the conception or design of the work; or the acquisition, analysis, or interpretation of data for the work; drafting the work or revising it critically for important intellectual content; agreement to be accountable for all aspects of the work in ensuring that questions related to the accuracy or integrity of any part of the work are appropriately investigated and resolved; final approval of the version to be published
